# Primary care clinicians’ perspectives on interacting with patients with gynaecological conditions: a systematic review

**DOI:** 10.3399/BJGPO.2023.0133

**Published:** 2024-03-20

**Authors:** Simon Briscoe, Jo Thompson Coon, G J Melendez-Torres, Rebecca Abbott, Liz Shaw, Michael Nunns, Ruth Garside

**Affiliations:** 1 Exeter PRP Evidence Review Facility, University of Exeter Medical School, St Luke’s Campus, University of Exeter, Exeter, Devon, UK; 2 NIHR Applied Research Collaboration South West Peninsula, University of Exeter Medical School, St Luke’s Campus, University of Exeter, Exeter, Devon, UK

**Keywords:** women’s health, systematic review, qualitative research, primary health care, gynaecological conditions

## Abstract

**Background:**

Studies have found that women with gynaecological conditions and symptoms do not feel listened to by primary care clinicians (PCCs). Less understood is whether PCCs perceive that there are challenges around listening to and interacting with this patient group.

**Aim:**

To understand PCCs’ perspectives on the challenges of listening to and interacting with women patients with gynaecological conditions and symptoms.

**Design & setting:**

Systematic review of English-language studies.

**Method:**

We searched ASSIA (Applied Social Sciences Index and Abstracts), CINAHL (Cumulative Index to Nursing and Allied Health Literature), Embase, HMIC (Healthcare Management Information Consortium), and MEDLINE from inception to July 2023. We also conducted forward and backward citation searches of included studies. Identified records were screened independently by two reviewers. Data extraction was undertaken by one reviewer and checked by a second. Quality appraisal used the Wallace checklist. 'Best-fit' framework synthesis was used to synthesise findings around themes that explored the challenges of patient–clinician interaction.

**Results:**

We identified 25 relevant articles. Perceived challenges associated with listening to and interacting with patients with gynaecological conditions and symptoms were identified at four ‘levels’: individual clinician level factors; structural and organisational factors; community and external factors; and factors related to gynaecological conditions. Interpretive analysis identified specific challenges relating to sociocultural factors affecting the consultation experience; the need for further education, training, or guidance for clinicians; factors affecting referral decisions; and factors related to service structure and organisation.

**Conclusion:**

PCCs acknowledge that empathy, respect, and attentive listening are important when interacting with women patients with gynaecological conditions and symptoms. However, these ideals are impeded by several factors.

## How this fits in

A substantial evidence base shows that women do not feel listened to by primary care clinicians (PCCs) when discussing their health concerns, particularly with respect to gynaecological conditions and symptoms. This is the first systematic review to address the challenges that PCCs report when listening to and interacting with women patients with gynaecological conditions and symptoms. The findings suggest that PCCs' interactions with women patients are impeded by their own limitations of knowledge and understanding, the structure and organisation of healthcare services, and the broader socio-cultural context. It is important to address these issues to support PCCs to provide appropriate treatment and support for women patients with gynaecological conditions and symptoms.

## Introduction

The Women’s Health Strategy for England (WHSE) has identified several priority areas where women’s health care needs improvement, including gynaecological conditions, fertility and pregnancy, mental health and wellbeing, cancer, healthy ageing, and long-term conditions.^1^ Of these, gynaecological conditions were the most frequently mentioned health condition by women responders to a survey undertaken to inform the WHSE on priorities for women’s health care (63% of responders).^1^ Key concerns included not feeling listened to by PCCs and length of time to diagnosis, both of which are commonly reported elsewhere in studies of women’s perspectives of gynaecological health care.^2–6^

Delays to accessing treatment and support for gynaecological conditions and symptoms can lead to both physical and mental ill-health.^7–11^ Policymakers and healthcare providers recognise the profound effects that this has on women’s health and wellbeing.^1,12,13^ The WHSE sets out measures to improve treatment and support for gynaecological conditions as a matter of high priority.^3^ What is less well-known is whether PCCs perceive that there are challenges around interacting with patients with gynaecological conditions and symptoms. However, there is an emerging body of qualitative research that explores the challenges of diagnosis and management for this patient group, within which data on listening to and interacting with patients are reported.^14–18^ The aim of this study was to understand PCCs’ perspectives on the challenges of listening to and interacting with women patients with gynaecological conditions and symptoms. The review was commissioned by the UK National Institute for Health Research (NIHR) Policy Research Programme to inform the WHSE.^1,19^

## Method

The method used for this systematic review followed established guidance.^20,21^ The protocol was registered on the Open Science Framework on 11 October 2021 (https://osf.io/2dw8n/).^22^ Reporting follows the Preferred Reporting Items for Systematic Reviews and Meta-Analyses (PRISMA) statement.^23^ Although in our protocol we initially set out to conduct a scoping review, we extended our analysis to a 'best-fit' framework synthesis to provide a richer analysis of the findings.^20^

### Study identification

We searched CINAHL (Cumulative Index to Nursing and Allied Health Literature), Embase, HMIC (Healthcare Management Information Consortium), MEDLINE, and ASSIA (Applied Social Sciences Index and Abstracts). We also conducted forward and backward citation searches of included studies. The bibliographic database search strategies are reproduced in Supplementary Box S1. Titles and abstracts were independently double-screened using inclusion criteria described in Box 1. Although we included studies if the participant population consisted of both PCCs and secondary care clinicians, we did not include data relating to solely secondary care clinicians from within these studies. Disagreements were resolved through discussion with a third research team member. Full texts were screened in the same way.

Box 1 **Inclusion criteria**

**Population**: Primary care clinicians, including (but not limited to) GPs or family doctors, nurse practitioners, and community pharmacists.

**Phenomenon of interest**: Interaction with patients with gynaecological conditions, including (but not limited to) endometriosis, menopause,

 menstrual disorders, polycystic ovary syndrome, or symptoms suggestive of these conditions.

**Context:** Primary care in World Bank high-income countries were included.^66^

**Study design**: Recognised qualitative methods including (but not limited to) thematic analysis, framework analysis, grounded theory,

 phenomenology. Surveys were excluded.

**Other:** Only English-language studies were included.

### Data extraction and quality appraisal

Data extraction included key study characteristics and identified themes. Data extraction and quality appraisal (using the Wallace checklist) was carried out by one reviewer and checked by a second.^24^

### Synthesis

We used 'best-fit' framework synthesis to synthesise the findings.^25^ This involved a two-stage process: first, the creation of a list of themes and sub-themes based on a pre-existing framework; second, an interpretive analysis in which the relationships between the themes and sub-themes are established and elaborated.^25^ We selected Dixon *et al* as containing the most well-developed framework among the included articles.^26^ Dixon *et al* explored PCCs’ perspectives on diagnosing and managing endometriosis.^26^ The four ‘level’ thematic framework presented in Dixon *et al* is shown in Table 1. (Additional sub-themes were presented underneath these themes.) Findings from included articles were coded against this framework, adapting and adding new themes as necessary.^25^ Coding was undertaken by one reviewer and checked by a second. Themes and sub-themes were tabulated and described narratively. The interpretative analysis sought to draw out how PCCs’ perspectives on listening to and interacting with women patients (as described in the themes and sub-themes) could be understood in terms of challenges to listening to and interacting with this patient group.

**Table 1.** Four level thematic framework

Theme	Explanation^a^
1. Individual clinician level factors	How decisions about patient care are informed by perspectives of PCCs on diagnosing and managing gynaecological conditions and symptoms in one-to-one consultation settings.
2. Structural and organisational factors	How the design and management of primary care settings affects the care provided, and how wider issues in secondary care affect primary care.
3. Community and external factors	How wider sociocultural issues and beliefs affect interactions between PCCs and patients.
4. Factors related to gynaecological conditions	How factors related to gynaecological conditions affect interactions between PCCs and patients, specifically where these factors were identified across multiple gynaecological conditions.

^a^We have changed language for ‘endometriosis’ as presented in Dixon *et al* to more generically ‘gynaecological conditions’ for the purpose of the broader scope of the present study.^26^ PCC = primary care clinician.

## Results

**Figure 1.**
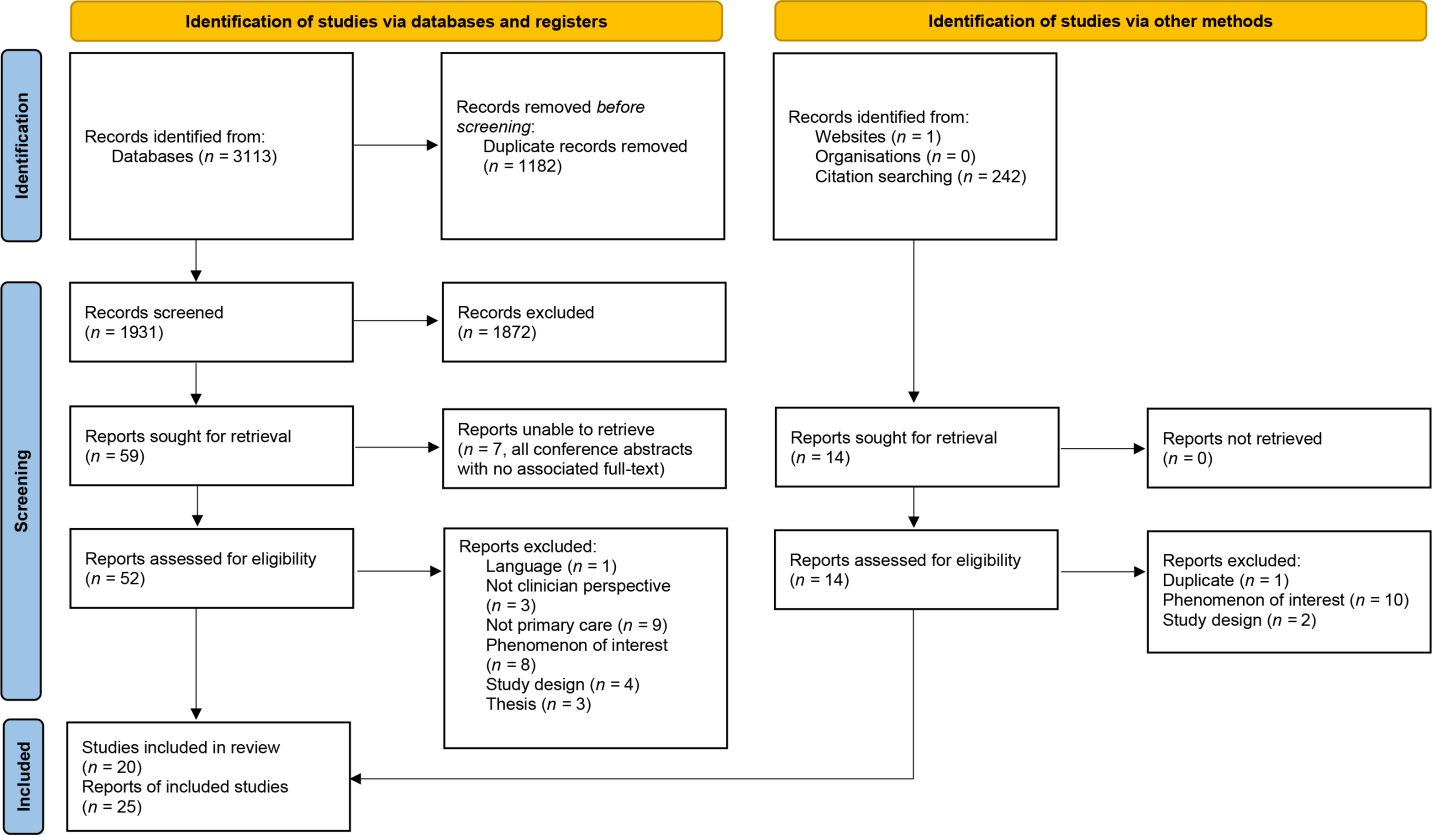
PRISMA flow diagram

Searches were conducted on 1 November 2021 and updated 3 July 2023 (see Supplementary Table S1). The screening process is depicted in Figure 1. The full list of studies excluded at full text is presented in Supplementary Table S2.

### Characteristics of included studies

Twenty-five articles published 1998–2023 were included^14–18,26–45^ (see Table 2 and Supplementary Table S3). Four studies reported that a proportion of participants had a special interest in gynaecology, which in some studies was achieved through purposive sampling.^17,29,41,43^ Other studies used purposive sampling to recruit participants who are routinely exposed to populations or diseases of interest.^30–32,35,37,40^ This sometimes meant that more female than male participants were included.^30,37^

**Table 2.** Study characteristics

Characteristic	Data (number of studies with data, %)
Type of primary care clinician	Community gynaecologist (1, 4%); GP (20, 80%); internist or family practitioner (2, 8%); nurse (4, 16%); pharmacist (2, 8%)
Condition	Chronic pelvic pain (2, 8%); endometriosis (8, 32%); infertility disease (1, 4%); menopause (6, 24%); menorrhagia (3, 12%); menstrual disorders (1, 4%); PCOS (3, 12%); premenstrual symptoms (1, 4%)
Country setting	Australia (7, 28%); The Netherlands (2, 8%); Norway (1, 4%); Sweden (2, 8%); UK (9, 36%); US (4, 16%)

PCOS = polycystic ovary syndrome.

### Quality appraisal

Overall, the articles were assessed as good quality (see Supplementary Table S4).

### Identified themes

PCCs’ perspectives on listening to and interacting with patients with gynaecological conditions or symptoms are described thematically within an adapted version of the four ‘level’ framework in Dixon *et al* in Supplementary Table S5.^26^ In addition, Supplementary Table S6 presents sub-themes that related to single gynaecological conditions only (organised separately under themes 5–10). To illustrate the findings of this stage of the analysis within the main text of this study, an abridged version of Supplementary Table S5 is presented in Table 3 showing the three sub-themes per theme with the most supporting studies.

**Table 3.** Themes and sub-themes with most supporting studies per theme (see Table S5 for full list of sub-themes)

Themes		Sub-themes(supporting studies, *n*)	Descriptions and supporting quotations
Individual clinician level factors	1.13^a^	Treating women with empathy and respect (*n* = 10)^14,16,26,28,34,38–41,44^	PCCs recognised the importance of empathising with how women feel. Respecting patients meant discussing symptoms and conditions in an open manner that was not dismissive of women’s ability to understand potential diagnoses and management.
	1.14	Understanding psychosocial impacts of gynaecological conditions (*n* = 13)^14,16,18,26,28,31,34,35,37–41,44^	PCCs recognised a need for an holistic approach to patient care, and showing an understanding of the psychological and social effects of a condition as well as the physiological effects.
	1.15	Using women’s subjective awareness of what is normal and abnormal to inform decision making (*n* = 10)^16,28,29,31,32,38,40,41,43,45^	PCCs described how they take into account women’s perceptions of what is normal or abnormal pain or bleeding. Abnormal pain was characterised as enough difference to interfere with patients’ daily living.^38^
Structural and organisational factors	2.2	Limited education for primary care clinical team (*n* = 6)^14,16,17,26,38,41^	PCCs reported that they did not receive much training on women’s health issues during their professional education or ongoing training. Limited education was raised as an issue for menopause, chronic pelvic pain and endometriosis, as well as generally for women’s health issues.
	2.5	Recognition of the importance of a multidisciplinary approach (*n* = 5)^16,17,28,34,38^	PCCs considered that a collaborative approach to working with other clinical specialists could improve the level of care that a patient receives.
	2.7	Unmanageable primary care doctor workload (*n* = 7)^14,15,18,26,27,32,36,44^	PCCs perceived that high levels of GP workload sometimes prevented them from doing more than the minimum required for their patients.
Community and external factors	3.3	Normalisation of symptoms in wider society and among clinicians (*n* = 6)^15,16,26,31,35,36,38,45^	PCCs perceived that symptoms of gynaecological conditions, including pain and heavy menstrual bleeding, are not always recognised by patients as outside of the normal range.
	3.4	Stigma or embarrassment of menstrual conditions and symptoms among patients (*n* = 8)^14,16,17,26,29,31,32,37^	PCCs perceived that there is stigma and embarrassment about menstrual conditions and symptoms. This can include cultural stigma surrounding menstruation and embarrassment about visible signs of bleeding.
	3.5	Web-based sources of accurate information are needed to correct misinformation (*n* = 6)^15,16,18,26,28,33,34^	PCCs noted that there was a preponderance of online misinformation about gynaecological conditions.^18,34^ PCCs would find it helpful to be able to signpost patients to reliable sources of online information.^16,26^
Factors related to gynaecological conditions	4.1	Gynaecological conditions can be difficult to definitively diagnose (*n* = 9)^17,18,26,28,29,34,37,41,43^	PCCs reported that some gynaecological conditions require multiple visits to see a clinician to assess the patients’ symptoms over time.
	4.3	Medicalisation of social phenomena or not believing there to be a physical issue (*n* = 7)^16,18,34,39,41,44,45^	PCCs considered that symptoms associated with gynaecological conditions could arise owing to psychological issues rather than physical issues, in particular, symptoms of endometriosis and chronic pelvic pain.^18,41,45^
	4.4	Need to follow a diagnostic hierarchy (*n* = 8)^16,17,26,34,36,38,41,44^	PCCs seek to exclude the most serious conditions before considering less serious or time-sensitive conditions. Once more serious conditions had been excluded, clinicians’ sense of urgency for a diagnosis was reduced if the symptoms were not severe.

^a^Subtheme numbers are the same as in Supplementary Table S5. PCC = primary care clinician.

### Interpretive analysis

Numbers in parentheses in this section refer to sub-themes in Supplementary Tables S5 and S6.

The identified themes and sub-themes suggest that clinicians recognise the need to treat women with empathy and respect (1.13), and acknowledge the psychosocial effects of gynaecological conditions and symptoms (1.14). However, given the findings of previous research, these perspectives have not translated into women feeling listened to. This section seeks to interpret PCCs’ perspectives on listening to and interacting with women patients in terms of challenges. To this end, we developed four interpretive themes related to the contexts in which primary care consultations take place:

- sociocultural factors affecting the consultation experience;
- the need for further education, training, or guidance for clinicians;
- factors affecting the decision to refer women, including obtaining a definitive diagnosis;
- factors related to service structure and organisations.

#### Theme 1: Sociocultural factors affecting the consultation experience

PCCs perceived that there remains stigma and embarrassment about gynaecological symptoms when discussing health concerns with patients, particularly among some minority ethnic groups (3.4). Furthermore, some clinicians perceived that patients considered that symptoms were part of normal life, which again was noted as prevalent within some minority ethnic groups (3.3). PCCs worried that these factors could lead to delays in women seeking appropriate care, or difficulties describing the experienced problem. However, researchers suggested in the analysis of their findings that clinicians might stereotype how minority ethnic groups view medical conditions rather than engage with individual patients (3.1).

There were differences in how male and female primary care doctors interact with women. With respect to menorrhagia, some male doctors reported relying solely on patients’ experience, feeling ill-equipped to do anything else (8.1). In contrast, female doctors indicated that they were more confident in combining in-depth exploration of patients’ experiences of menstrual bleeding with clinical judgement about the abnormality of the symptoms (8.1). More generally, some PCCs, who were often male, unhelpfully expected patients to comply rather than engage with care, viewing them as ‘good’ or ‘bad’ depending on how well they followed advice (1.16).

While some PCCs preferred patients who came to consultations with a clear idea of what they want (1.1), others thought information gleaned online might be inaccurate, leading to beliefs that were difficult to challenge (3.5). Accurate sources of online information were considered necessary to help patients understand their symptoms and for PCCs to be able to direct patients (3.5). Sometimes lifestyle and psychological factors, such as obesity, were considered as potential causes of symptoms that needed to be addressed before the patient would see an improvement in health (4.3). However, PCCs also felt that patients with polycystic ovary syndrome (PCOS) were reluctant to accept that there were no easy solutions (6.1) and considered that patients with chronic pelvic pain would disengage with primary care if they felt that they were not receiving appropriate treatment (9.2).

#### Theme 2: Need for further education, training, or guidance for clinicians

PCCs sometimes lacked sufficient knowledge of gynaecological conditions (1.6) owing to limited education (2.1) and guidelines (4.2), or infrequent exposure to these conditions (1.5) (especially among male clinicians). Understanding the difference between normality and pathology was considered an area that needed improvement in guidance across multiple conditions, including endometriosis, menopause, and menorrhagia (4.2). For example, understanding the difference between dysmenorrhoea and symptoms of endometriosis was considered challenging (4.1) and not clearly defined in guidance (4.2). Conversely, it was deemed unrealistic to become familiar with the extent of guidance across all potential health conditions that women experience (6.2). The extent of guidance combined with its perceived shortcomings meant that navigating and implementing guidance was challenging.

PCCs also reported struggling to find solutions for patients dissatisfied with care, particularly patients with long-term conditions (4.6). Chronic pelvic pain symptoms were often long-term and had no clear biomedical explanation, which was noted by GPs as particularly challenging for practice nurses (9.4). In particular, it was observed by GPs that practice nurses mainly carry out tests such as swabs, and have more limited skills for dealing with patients who may be somatising. However, despite the challenges entailed, practice nurses considered they could play a more significant role than they currently do with this patient group as they are often the first point of contact (9.4).

#### Theme 3: Diagnosis and decisions to refer women

Decisions about when to refer presented challenges for PCCs both from the perspective of making an appropriate judgement, and managing patient expectations of what constitutes a satisfactory outcome for consultation. It was noted that many gynaecological conditions are difficult to definitively diagnose, and some diagnoses require tests only obtainable through referral (4.1). However, referrals were not always made, even if there were symptoms that could justifiably lead to a referral. Sometimes PCCs did not refer because of concerns about potential adverse effects of invasive diagnostic tests, which meant they only referred if they considered there was a high probability of pathology (1.3). For example, younger women with symptoms of endometriosis were sometimes not referred owing to similarities with normal period pain (1.3). Overall, younger women were seen as less likely to have serious pathology and were less likely to be referred (1.16).

Some PCCs did not think a diagnosis was necessary if the symptoms could be adequately controlled in primary care (1.7) or would not affect treatment (1.12). For example, PCCs reported that patients who had been referred were sometimes told to focus on symptom control rather than investigation, which is something that they could do themselves (1.12). There was also reluctance to give patients a ‘label’ too early, particularly if this could cause patient anxiety (1.8) and concern to avoid over- or mis-diagnosis, which could lead to failure to treat the actual problem (4.5). Strategies for deciding when to refer included following a ‘diagnostic hierarchy’, which excluded red flags (such as cancer) after which PCCs had a reduced sense of urgency to investigate other conditions (4.4).

Perceived pressures on the healthcare system in secondary care might also influence decisions to refer (2.4). However, women who did not have English as a first language, those not wanting to be examined by a male clinician, or where infertility was a concern, might be more likely to be referred (3.2). More engaged and proactive women were also thought more likely to get referred (1.1).

#### Theme 4: Factors related to service structure and organisations

Challenges for clinician–patient interaction were also apparent in the structure and organisation of healthcare services. These included high GP workload and limited consultation time, potentially having a detrimental effect on quality of care (2.7). A lack of continuity of care was also perceived as a challenge, particularly where the need to build rapport with patients was seen as critical to effective communication and understanding (2.6).

## Discussion

### Summary

Our findings show that PCCs perceive that attentive listening is important when discussing gynaecological conditions and symptoms with patients. However, our findings also show that sociocultural factors can make it challenging to discuss symptoms with some patient groups, and can create different understandings of what constitutes pathology. Furthermore, gaps in understanding are not always covered in guidance, and factors influencing the decision to refer or diagnose gynaecological conditions can lead to a mismatch of expectations between PCCs and women patients. Structural issues present challenges for building rapport with patients.

### Strengths and limitations

This review includes evidence on clinician–patient interaction relating to a variety of gynaecological conditions and symptoms. However, although we were able to identify data on listening to and interacting with women patients, in most studies data were inferred from wider discussion of the diagnosis and management of gynaecological conditions. Furthermore, differences in participant recruitment approaches in the included studies may have led to different perspectives being reported (for example, specialist versus non-specialist participants; male versus female participants). Sometimes this is drawn out in the findings of this review (for example, differences in the perspectives of male and female clinicians) but there is scope for more nuance in considering the differences between specialist and non-specialist PCCs.

### Comparison with existing literature

Several systematic reviews to date focus on patients’ experiences of interacting with clinicians with respect to gynaecological conditions, including PCCs.^46–53^ These reviews draw attention to women not feeling listened to in consultation settings. However, to the best of our knowledge, ours is the first systematic review to address the challenges that PCCs’ report when listening to and interacting with this patient group. Similar findings about women’s experiences of health care more broadly have also been reported; for example, there is evidence that women experiencing chronic pain feel dismissed by clinicians.^54–56^ Furthermore, several studies show that men and women presenting with the same or similar symptoms are treated differently, with women more frequently receiving misdiagnosis or mismanagement of symptoms.^57–61^ This suggests that negative experiences of care experienced by women with gynaecological conditions or symptoms are part of a wider gender-related problem. Nonetheless, our review reveals specific challenges around listening to and interacting with this patient group.

### Implications for research and practice

The complexity of the different factors that contribute to the challenges that PCCs experience in this area make it unlikely that there are simple solutions. Training and guidance are likely to be important in improving women patients’ experiences in primary care, and should address the importance of empathetic, respectful, and attentive communication, as well as the challenges of diagnosing and managing gynaecological conditions and symptoms. Studies show that improved listening and communication can improve trust in the clinician–patient relationship.^62,63^ However, our findings show there are also structural and organisational issues that need addressing. One way of achieving longer consultation times may be to more fully integrate a wider variety of healthcare professionals into routine consultation work as proposed in the UK *NHS Five Year Forward View*.^64^ Furthermore, any proposed changes to improving the timeliness and accuracy of diagnoses of gynaecological conditions and symptoms should take into account the view that diagnosis is not always the best outcome, even if symptoms are present.^65^

Sociocultural issues are difficult to address, particularly perceptions in the wider society, which are largely outside of clinicians’ and policymakers’ control. In particular, our findings show that patients in minority ethnic groups pose specific sociocultural challenges.^15,31^ More research in this area would be valuable. Calls have been made to implement public awareness campaigns that challenge the stigma of gynaecological conditions, and enhance the visibility of conversations about them, which we consider an important part of the solution.^9,11^ Women patients’ widely held preference to see female PCCs, however, may mean that although compulsory training on women’s health for all PCCs might improve the basic level of knowledge and awareness, male clinicians with less exposure to gynaecological conditions will still likely provide a different standard of care to female clinicians.^1^

Finally, further primary research is required that explicitly sets out to investigate the perspectives of PCCs on listening to and interacting with women patients.^1^

## Data

The data that support the findings of this study are available from the corresponding author upon reasonable request.
